# Cellular Vulnerability and Concomitant Pathology in Atypical AD

**DOI:** 10.1002/alz70855_099052

**Published:** 2025-12-23

**Authors:** Lea T. Grinberg

**Affiliations:** ^1^ University of São Paulo Medical School, São Paulo, São Paulo, Brazil; Memory & Aging Center, Department of Neurology, University of California in San Francisco, San Francisco, CA, USA

## Abstract

**Background:**

Although individuals meeting neuropathological criteria for Alzheimer's disease (AD) may present with distinct clinical syndromes, the basis for these atypical manifestations remains incompletely understood. Traditional views have pointed to comorbid pathologies (e.g., TDP‐43, Lewy bodies) as drivers of clinical heterogeneity. However, recent work from our group suggests that these additional pathologies do not significantly influence atypical AD phenotypes. Instead, growing evidence—including our own—points to selective neuronal vulnerability, particularly to tau, indicating that AD could be a pathogenic convergence of multiple disease processes rather than a single entity.

**Method:**

To clarify the foundations of atypical AD manifestations, we conducted a series of studies using postmortem brain samples from clinically well‐characterized cases. We combined classical neuropathological evaluations (e.g., histopathology, immunohistochemistry) with single‐nucleus RNA sequencing (snRNA‐seq), transcriptomics, and proteomics analyses to identify neuronal subpopulations most susceptible to tau‐driven pathology and to delineate potential molecular pathways distinguishing typical from atypical AD. We also examined subcortical regions involved in sleep‐wake regulation, hypothesizing that divergent tau deposition in these areas may underlie the varied sleep profiles observed in AD variants.

**Result:**

Our findings indicate that comorbid neuropathologies do not significantly correlate with atypical presentations. Instead, specific neuronal populations, defined by snRNA‐seq signatures, display heightened vulnerability to tau pathology in atypical AD cases. This susceptibility appears linked to unique molecular pathways that may shape different clinical trajectories. Furthermore, our investigation of subcortical sleep‐wake control centers reveals that variant‐specific tau burden aligns with distinct sleep disturbances, underscoring the importance of region‐ and cell‐specific mechanisms in determining clinical phenotypes.

**Conclusion:**

Atypical AD variants offer a valuable window into the complex interplay between neuropathological processes and clinical outcomes. By integrating classical neuropathology with advanced multi‐omics, we capture the biological diversity of this heterogeneous disease more comprehensively. Our data support a model in which AD represents a convergence of multiple pathogenic processes rather than a singular disease entity. This perspective will guide future diagnostic, prognostic, and therapeutic approaches, paving the way for more personalized strategies to manage the broad spectrum of AD presentations.

